# Arthroscopically assisted reduction and internal fixation of a femoral anterior cruciate ligament osteochondral avulsion fracture in a 14-year-old girl via transphyseal inside-out technique

**DOI:** 10.1007/s11751-013-0175-6

**Published:** 2013-09-04

**Authors:** Ronny Langenhan, Matthias Baumann, Bernd Hohendorff, Axel Probst, Per Trobisch

**Affiliations:** 1 Klinik für Orthopädie, Unfall- und Handchirurgie, Hegau-Bodensee-Klinikum Singen, Virchowstr. 10, 78224 Singen, Germany; 2 Berufsgenossenschaftliche Unfallklinik, Eberhard-Karls-Universität Tübingen, Schnarrenbergstr. 95, 72076 Tübingen, Germany; 3 Elbe Kliniken Stade Buxtehude, Abteilung für Hand-, Ästhetische und Plastische Chirurgie, Bremervörder Straße 111, 21682 Stade, Germany; 4 Orthopädische Universitätsklinik Magdeburg, Leipziger Str. 44, 39120 Magdeburg, Germany

**Keywords:** Anterior cruciate ligament, Skeletally immature, Femoral avulsion fracture, Arthroscopic reduction and internal fixation

## Abstract

Femoral avulsion fracture of the anterior cruciate ligament (ACL) in children and adolescents is rare, and its arthroscopic treatment is even more so. A femoral avulsion fracture of the ACL of a 14-year-old girl was arthroscopically reduced and fixed by a Kirschner wire (K-wire) via an inside-out technique. A 1.4-mm K-wire was drilled inside-out into the osseous defect of the lateral femoral condyle under arthroscopic visualization. The avulsed fragment was reduced and then drilled retrograde by the wire. After bending the intra-articular visible end of the K-wire by a knot pusher, the fragment was gently fixed by pulling the wire from outside. At 24 months, both knee stability and range of motion were the same in the operated and the healthy opposite leg. Magnetic resonance imaging evaluation and conventional radiographs showed an intact ACL without detectable disturbance in the growth plate. Only seven cases of a proximal avulsion of the ACL in children and adolescents have been published. Six were treated by open reduction and internal fixation, one by arthroscopic reduction without fixation.

## Introduction

Anterior cruciate ligament (ACL) injuries in children and adolescents are rare, comprising about 0.5 % of all ACL tears [1]. Bone avulsions are more frequent due to weaker epiphyseal insertion compared to strong elastic ligaments [2]. A tibial avulsion of the ACL is the most common form and also the most common osseous lesion in knee injuries of the growing skeleton [3]. In contrast, femoral osteochondral avulsion fracture of the ACL in children is a rarity. To our knowledge, only seven cases have been reported so far [2–8].

We present a 14-year-old girl with a femoral osteochondral avulsion fracture of the ACL, which was arthroscopically reduced and fixed by a Kirschner wire (K-wire) via an inside-out technique.

## Patient and methods

**Fig. 1:**
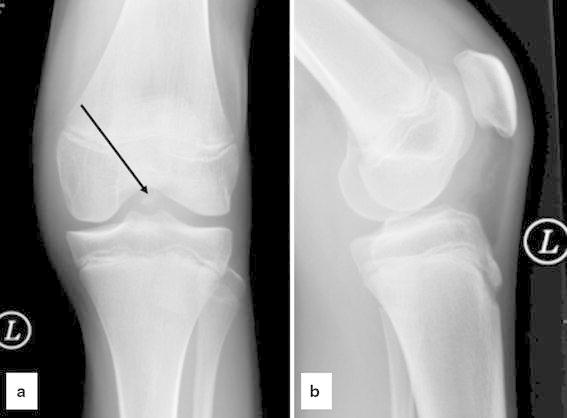
Anteroposterior (**a**) and lateral (**b**) view of the left knee with a femoral osteochondral avulsion fracture of the ACL (*black arrow*)

**Fig. 2:**
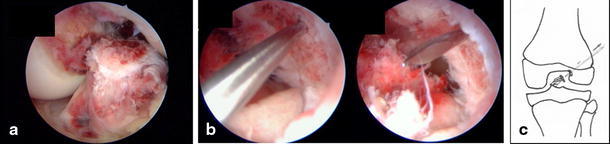
Arthroscopic visualization of the 1.4-mm K-wire (**a**), drilled inside-out into the center of the osseous defect of the lateral femoral condyle. Schematic of surgical technique (**b**)

**Fig. 3:**
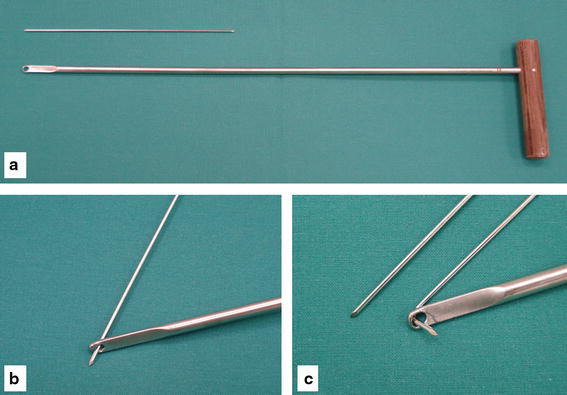
Knot pusher and 1.4-mm K-wire (**a**). Bending technique of the K-wire (**b**, **c**)

**Fig. 4:**
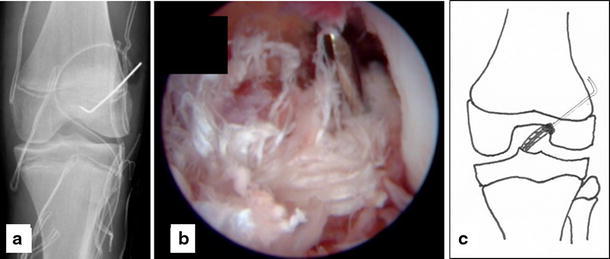
Postoperative anteroposterior radiograph of the left knee with the K-wire, distal end bent (**a**). Arthroscopic view with reduced femoral avulsion fragment fixated by the K-wire (**b**). Schematic of surgical technique (**c**)

**Fig. 5:**
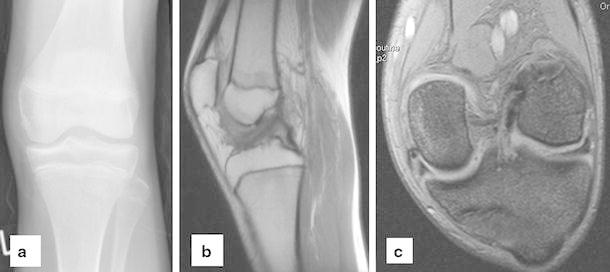
Anteroposterior radiograph of the left knee (**a**), sagittal MRI (**b**), and coronal MRI (**c**) at 24-month follow-up with intact ACL and without detectable disturbance of the growth plate of the distal femur

A 14-year-old girl suffered a distortion of her left knee during school sports. At initial investigation, passive motion of the knee was restricted due to pain and swelling (extension/flexion, 0/20/70°). The Lachman and pivot shift tests were positive. Evaluation of conventional radiographs (Fig. 1) and magnetic resonance imaging (MRI) showed a femoral osteochondral avulsion fracture of the ACL without injury of other structures. An arthroscopic reduction and internal fixation was performed. A 1.4-mm K-wire was drilled inside-out into the center of the osseous defect of the lateral femoral condyle under arthroscopic visualization (Fig. 2). The avulsed fragment was reduced anatomically and then drilled retrograde by the K-wire, while the knee was positioned in 20° of flexion. After bending the visible intra-articular end of the K-wire by a knot pusher, the fragment was gently fixed by pulling the wire from outside. Figure 3 shows the instruments and bending technique of the K-wire. The proximal end of the K-wire was cut under the fascia of the M. vastus lateralis (Fig. 4). After operation, the knee was immobilized in a brace in 20° of flexion for 6 weeks. Three months postoperatively, the K-wire was removed by pulling it gently from the proximal end. The bent intra-articular part of the wire straightened upon pulling, and the whole wire was removed without any complication. An elective diagnostic arthroscopy confirmed a stable ACL. Upon follow-up at 24 months, clinical examination revealed no effusion and no passive motion deficit (extension/flexion, 5/0/150°, comparable to the opposite healthy leg). The knee ligaments were stable (negative Lachman, reverse pivot shift, and pivot shift tests). Neither patellofemoral nor medial or lateral compartment crepitation was detectable. The girl performed a one-leg hop three times for both legs without limitation. She also had no limitations in sports activities. Both MRI evaluation and conventional radiographs showed an intact ACL without detectable disturbance in the growth plate of the distal femur (Fig. 5). Grade A was noted using the IKDC Knee Examination Form [9].

## Discussion

To our knowledge, this is the first case report of an adolescent with a femoral osteochondral avulsion fracture of the ACL that was arthroscopically reduced and fixed by a K-wire via an inside-out technique.

ACL injuries in skeletally immature athletes have significantly increased, primarily due to increased awareness of these injuries within this younger athletic population. There is growing evidence to suggest that ACL rupture in children is more common than previously thought and a poor outcome is associated with conservative management [3, 10]. The site of rupture in children and adolescents is predominantly tibial avulsion, but midsubstance tears have also been reported [3]. However, femoral osteochondral avulsion fracture of the ACL in children and adolescents is a rarity.

Corso and Whipple [4] reported the case of a 3-year-old boy with swelling of the left knee, which was unresolved after 10 days. He could not place weight on the knee or extend it beyond 45° and had significant pain with passive extension. Arthroscopy revealed that his ACL appeared to have been sheared from its cartilaginous attachment to the lateral femoral condyle. The attachment to the femur was only debrided and abraded, and the knee was then immobilized in extension in a long leg cast after reducing the proximal end of the ACL.

Eady et al. [5] repaired an osteochondral avulsion of the femoral origin of the ACL also through a medial parapatellar incision with two sutures, which were pulled through two epiphyseal 3.2-mm drill holes in the lateral femoral condyle. Before starting with assisted active and passive exercises, the knee was immobilized with a long leg cast in 30° of flexion for 6 weeks after the operation. At the 15-month follow-up, the 7-year-old girl was free of symptoms without instability and normal roentgenograms.

Kawate et al. [6] fixed a proximal cartilaginous avulsion fracture of the ACL with a 0.5-mm stainless steel wire passed through two 1.8-mm drill holes crossing the physis after entering the joint through a medial parapatellar incision. The knee was immobilized in a 30° flexion in an above-the-knee cast, and the metal was removed after 5 months. Thirteen years later, the 16-year-old boy had no problems. The radiological findings were normal.

Robinson and Driscoll [2] reported a case of a simultaneous osteochondral avulsion of the femoral and tibial site of the ACL in a 13-year-old boy. The knee was explored through a long, medial parapatellar incision. The tibial avulsion fragment was tied with sutures through epiphyseal drill holes. The femoral site of the ACL was repaired by passing sutures through two drill holes across the physis to the lateral metaphyseal area of the femur. The patient’s knee was immobilized in a long cast for 7 weeks. At 1-year follow-up, the patient had no symptoms in the knee and was fully active.

Other authors also treated femoral osteochondral avulsion fractures of the ACL using parapatellar incisions and pullout sutures through drill holes [2, 3, 7].

Expert opinion regarding experience with the management and complications of pediatric ACL injuries was studied by surveying members of the Herodicus Society and the ACL Study Group [11]. There was large practice variation in initial management and ACL reconstruction technique. Fifteen cases of growth disturbance were reported: eight cases of distal femoral valgus deformity with arrest of the lateral distal femoral physis, three cases of tibial recurvatum with arrest of the tibial tubercle apophysis, two cases of genu valgum without arrest, and two cases of leg length discrepancy. Associated factors included fixation hardware across the lateral distal femoral physis. Based on this experience, the authors recommend a guarded approach to ACL reconstruction in the skeletally immature patient with careful attention to technique and follow-up.

Koman and Sanders [12] reported the case of a 14-year-old boy with a ruptured ACL, which was reconstructed arthroscopically with a double-stranded semitendinosus graft. Two years later, a progressive valgus deformity with a prematurely completely fused distal femoral growth plate was observed. The authors concluded that ACL reconstruction with bone plugs or hardware crossing the growth plates would be contraindicated and should be delayed until the patient reaches skeletal maturity.

Mäkelä et al. [13] operated 5-week-old rabbits. In one group, a 2-mm drill hole was made in the intercondylar portion of the right femur across the central portion of the growth plate up to the diaphysis, while in the other group a similar drill hole of 3.2 mm was made. At 3, 6, 12, and 24 weeks after operation, specimens from the growth plates of both femora were analyzed using radiographic, microradiographic, histological, and histomorphometric techniques. The authors found that destruction of 7 % of the cross-sectional area of the growth plate caused permanent growth disturbance and shortening of the femur. Disturbance of the growth plate of the distal femur condyle by the K-wire via inside-out technique described in our case was negligible. At 12-month follow-up, both MRI evaluation and conventional radiographs showed an intact growth plate of the distal femur.

Bonin et al. [14] described a simple technique for arthroscopic fixation of tibial intercondylar eminence avulsion fractures using a folded surgical pin, which is comparable with our technique. A 1.8-mm K-wire was drilled through the guide from the proximal tibia into the reduced fragment. It was bent on its end into the joint with a strong needle case. The wire was then pulled back until good fragment compression to the tibia appeared with the wire straightening. The other side was bent on the anterior tibial cortex and cut. The arthroscopic fixation allowed elastic compression fragment stabilization that authorized early weight-bearing and rehabilitation programs. The material was extracted by traction after 6 months.

Our case suggests that arthroscopic reduction and internal fixation by a K-wire via inside-out technique provides an accurate and effective treatment for ACL avulsion fractures, which could help to avoid chronic ligamentous laxity and decrease the morbidity associated with open procedures [15].

## Conclusions

Arthroscopic reduction and internal fixation by a K-wire via an inside-out technique is a simple and effective procedure for ACL femoral osteochondral avulsion fracture in a skeletally immature athlete.
